# Providing dental insurance can positively impact oral health outcomes in Ontario

**DOI:** 10.1186/s12913-020-4967-3

**Published:** 2020-02-17

**Authors:** Nevena Zivkovic, Musfer Aldossri, Noha Gomaa, Julie W. Farmer, Sonica Singhal, Carlos Quiñonez, Vahid Ravaghi

**Affiliations:** 1 Dental Public Health, Faculty of Dentistry, University of Toronto, Toronto, Canada; 2 School of Dentistry, University of Birmingham, Birmingham, England

**Keywords:** Dental care, Oral health, Insurance, Dental insurance, Dental utilization, Dental visits, Dental outcomes, Privately financed care, Canada

## Abstract

**Background:**

Universal coverage for dental care is a topical policy debate across Canada, but the impact of dental insurance on improving oral health-related outcomes remains empirically unexplored in this population.

**Methods:**

We used data on individuals 12 years of age and older from the Canadian Community Health Survey 2013–2014 to estimate the marginal effects (ME) of having dental insurance in Ontario, Canada’s most populated province (*n* = 42,553 representing 11,682,112 Ontarians). ME were derived from multi-variable logistic regression models for dental visiting behaviour and oral health status outcomes. We also investigated the ME of insurance across income, education and age subgroups.

**Results:**

Having dental insurance increased the proportion of participants who visited the dentist in the past year (56.6 to 79.4%, ME: 22.8, 95% confidence interval (CI): 20.9–24.7) and who reported very good or excellent oral health (48.3 to 57.9%, ME: 9.6, 95%CI: 7.6–11.5). Compared to the highest income group, having dental insurance had a greater ME for the lowest income groups for dental visiting behaviour: dental visit in the past 12 months (ME highest: 17.9; 95% CI: 15.9–19.8 vs. ME lowest: 27.2; 95% CI: 25.0–29.3) and visiting a dentist only for emergencies (ME highest: -11.5; 95% CI: − 13.2 to − 9.9 vs. ME lowest: -27.2; 95% CI: − 29.5 to − 24.8).

**Conclusions:**

Findings suggest that dental insurance is associated with improved dental visiting behaviours and oral health status outcomes. Policymakers could consider universal dental coverage as a means to support financially vulnerable populations and to reduce oral health disparities between the rich and the poor.

## Introduction

In North America, there is a clear income gradient in accessing dental care, with higher-income earners more likely to visit the dentist [1, 2]. Approximately one-in-five Canadians report financial barrier to dental care [3]. Canada ranks highest in income inequalities in visiting the dentist, compared to eighteen Organization for Economic Cooperation and Development (OECD) countries [4]. Compared to eleven other Commonwealth countries, Canada ranked second highest in the proportion of individuals who skipped dental care in the past year due to cost [5].

Access to dental care is affected by systems-level decisions in the provision of services and the degree of dental insurance coverage [6]. These factors can have far-reaching consequences on oral health outcomes and inequalities on a population level [7–10]. In contrast to many high-income European countries, Canada has a predominantly private dental care system. In Canada, the majority of dental care services are paid through out of pocket payments and private dental insurance (employment-based or individually purchased). Canada also has high per capita spending on dental care, third highest among OECD countries [11]. We would expect that having any type of dental insurance coverage may play a significant role in oral health inequalities.

Canadians who cannot afford dental care are also more likely to have worse oral health outcomes, leading to a greater need for dental treatment [12]. Further, it has become clear that it is not just unemployed or low income earning Canadians who have difficulty affording and accessing dental care, but also middle income earning adults [13–15]. Canada’s dental care system shows evidence of the ‘inverse care law,’ where those who need treatment the most are the ones who cannot afford it and the least likely to receive it [16].

The World Health Organization advocates for universal dental coverage as a means to ensuring everyone can access medically necessary care [17]. Dental coverage removes the cost barriers that prevent individuals from accessing care. A systematic review and meta-analysis found that dental insurance has a significant effect on increasing utilization in dental care [18]. Studies from Canada, the United States, and Australia have shown that people with dental insurance report greater dental service utilization and lower rates of unmet need [1, 12, 19–22]. In addition, there is growing policy and civil society interest in universal dental care coverage in Canada [23, 24].

The use of marginal effects models (ME) in the Canadian dental insurance market is a novel approach to report the population-level impact of insurance in this context. These models provide a straightforward interpretation of the results with more direct policy implications compared to the estimation of relative differences between groups [25, 26]. Results from ME models can provide insight for policymakers on the potential impact of a universal dental coverage scheme on dental care outcomes at the population level.

Using data from Ontario, Canada’s most populated province, our aims were: (i) to learn about the potential impact of dental insurance on various oral health related outcomes, and (ii) to describe the population-level impact of having dental insurance on these outcomes. Our objectives were: (i) to estimate the impact of dental insurance on dental visit behaviour and oral health status outcomes in Ontario; and (ii) to compare the impact of insurance on these outcomes across income, education and age subgroups; in other words, we wanted to see whether some groups are more sensitive to insurance coverage than others. While our first objective has indeed been addressed in prior research, to the best of our knowledge there has not been any research exploring the differential impact of dental insurance on socio-demographic groups.

## Methods

### Data source

We obtained data from the nationally representative 2013–2014 Canadian Community Health Survey (CCHS). This study includes 42,553 Ontarians, and incorporates sample weights to produce provincially representative results for a population of 11,682,112. Public use microdata files were accessed through the University of Toronto. This research uses non-identifiable secondary data that are publicly accessible. According to Article 2.4 of the Tri-Council Policy Statement: Ethical Conduct for Research Involvement Humans, research ethics board approval is not required for this study [27].

The CCHS is a population-based telephone survey that collects data on individuals 12 years or older in Canada’s provinces and territories. Individuals living on Indian Reserves and Crown Lands, institutions such as long term care facilities, full-time members of the Canadian forces, and some remote regions do not constitute the sampling frame. Further details on the design and sampling characteristics of the CCHS are outlined elsewhere [28].

### Outcome variables

We report the impact of insurance on two dental visiting behavior outcomes and two oral health status outcomes. The dental visiting behavior outcomes include: visiting a dentist in the past 12 months, and visiting a dentist only for emergencies. Visiting the dentist in the past 12 months was defined as whether an individual visited the dentist for any reason, at least once, in the past 12 months. In the survey, respondents were asked: “Do you usually visit the dentist more than once a year for check-ups, about once a year for check-ups, less than once a year for check-ups, or only for emergency care.” Visiting a dentist only for emergencies was dichotomized as individuals who visit only for emergency care versus those who visit for check-ups. The oral health outcomes include: very good or excellent self-reported oral health (SROH) and tooth loss due to decay or gum disease. SROH is measured on a five-point scale from poor to excellent. In this survey, we grouped very good and excellent as our outcome of interest. SROH data is often collected in large population surveys, providing a good summary measure of people’s perception of their oral health [29]. Tooth loss due to decay or gum disease is defined as tooth extraction due to decay or gum disease by a dentist in the past 12 months. We restricted this outcome to a subgroup of individuals who visited a dentist in the past 12 months and who reported having at least one of their own teeth at the time of survey completion.

### Covariates

Covariates of interest included: age, sex, dental insurance, household income decile, highest level of household education, self-perceived general health, geographic region, and having at least one tooth. We categorized age into five groups: 12–17, 18–34, 35–49, 50–64 and 65 and older. Dental insurance includes any coverage (public and private) that offsets the cost of dental services partially or completely. Household income is a proxy for socioeconomic status and reflects an individuals’ ability to afford dental care. Household income deciles were computed at the provincial level as the ratio of household income divided by the low-income cut-off of the individual’s neighbourhood. Statistics Canada imputed missing income data using a complex modelling method of near neighbour imputation [30]. We collapsed deciles into quintiles for analysis. Household education served as an indicator of health literacy level. General health status or having at least one tooth reflect current health status measures that may influence the potential need for dental treatment (i.e. poor general health may indicate more help is needed to maintain oral health). Geographic region was grouped into seven peer groups as outlined by Statistics Canada in order to adjust for population demographics, living conditions and working conditions [31].

### Statistical analysis

Survey weighted proportions were calculated for baseline characteristics. The proportion of individuals with dental insurance and their 95% confidence intervals (CI) in each group is reported.

Marginal effects (ME), using the average ME approach described by Onukwugha and colleagues, were used to calculate the change in each of the outcomes based on a unit change in covariate variable from the reference category (26). ME are regression-based estimates that represent the absolute change in outcome between two groups. For example, when comparing dental visits between individuals with and without insurance (reference group), a ME of 40 would indicate a 40 percentage point increase in reporting an annual dental visit for individuals with compared to those without dental insurance. Further information on the use and interpretation of marginal effects is described elsewhere [25]. For this paper, all four outcomes were modeled using logistic regression and adjusted for the aforementioned covariates. To address the first objective, we report the unadjusted and adjusted ME of insurance for each outcome. To address the second objective, we report the adjusted ME of insurance status on each outcome for each strata of household income, household education, and age group. This allows us to assess whether some strata are more sensitive to dental insurance than others.

We assumed data was missing at random (MAR), so we used multiple imputation with chained equations (MICE) to reduce risk of bias [32]. The original dataset had income imputation completed by Statistics Canada as described previously. Had we used this dataset, we would have had 91.5% data completion in the full analysis, and 95.9% in our subpopulation analysis. However, we decided to impute the remaining missing variables using MICE. All analyses were conducted using STATA/IC 13.1.

## Results

In our sample, approximately two-thirds of individuals report having dental insurance (66.2, 95%CI: 65.3 to 67.1) (Table 1) and the majority have at least one or more of their own teeth (95.3, 95%CI: 95.0 to 95.7). The proportion of individuals with dental insurance varies significantly amongst all sociodemographic characteristics reported, with the exception of sex. Supplementary data on the association between outcomes and select covariates (dental insurance, income, education and age groups) are reported in Additional files 1 and 2.

**Table 1:** Baseline characteristics of Ontarians in the 2013–2014 Canadian Community Health Survey. Proportions with 95% confidence intervals are reported

	Proportion in population	Proportion with insurance
Age		
12 to 17	8.2 (7.7–8.6)	78.3 (75.7–80.9)
18 to 34	26.4 (25.6–27.2)	66.2 (64.4–68.0)
35 to 49	24.0 (23.1–24.9)	75.9 (73.9–77.9)
50 to 64	24.3 (23.5–25.2)	72.2 (70.5–74.0)
65 years or more	17.1 (16.6–17.6)	38.2 (36.7–39.8)
Sex		
Male	48.9 (48.0–49.8)	67.1 (65.8–68.4)
Female	51.1 (50.2–52.0)	65.3 (64.1–66.5)
Dental Insurance		
No	33.8 (32.9–34.7)	NA
Yes	66.2 (65.3–67.1)	NA
Household income quintile		
Lowest	20.0 (19.2–20.8)	44.2 (41.9–46.6)
Lower middle	20.0 (19.3–20.8)	56.2 (54.1–58.3)
Middle	20.0 (19.3–20.8)	68.6 (66.7–70.5)
Upper middle	20.0 (19.3–20.8)	78.9 (77.3–80.5)
Highest	19.9 (19.2–20.6)	83.1 (81.7–84.5)
Highest household education		
< Than Secondary	5.8 (5.4–6.2)	34.2 (31.1–37.3)
Secondary graduate	12.5 (11.9–13.1)	55.7 (53.3–58.2)
Some post secondary	3.3 (2.9–3.7)	59.6 (53.7–65.6)
Post secondary graduate	78.4 (77.7–79.2)	70.5 (69.5–71.5)
Self-reported general health		
Excellent	21.0 (20.3–21.8)	70.5 (68.7–72.3)
Very good	38.5 (37.6–39.4)	70.4 (69.1–71.8)
Good	28.7 (27.9–29.6)	62.7 (61.0–64.5)
Fair	8.4 (7.9–8.9)	55.3 (52.4–58.3)
Poor	3.4 (3.0–3.7)	47.1 (41.7–52.5)
Has at least one of own teeth		
No	4.7 (4.3–5.0)	36.0 (32.3–39.7)
Yes	95.3 (95.0–95.7)	67.7 (66.8–68.6)

There are 42,553 individuals sampled representing 11,682,112 Ontarians. Missing data was imputed to provide population level estimates

### Objective 1: the impact of dental insurance on dental visiting behaviours and oral health status outcomes

Table 2 shows the association between dental insurance status with dental visiting behaviours and oral health outcomes (unadjusted and adjusted MEs). Overall, dental insurance has a positive impact on visiting a dentist in the past 12 months (ME: 22.8, 95% CI: 20.9–24.7), and reporting very good or excellent SROH (ME: 9.6, 95% CI: 7.6–11.5). Dental insurance had a negative impact on visiting a dentist only for emergencies (ME: -19.1, 95% CI: − 20.9 to − 17.4), or experiencing tooth loss due to decay and gum disease (ME: -1.5, 95% CI: − 2.7 to − 0.3).

**Table 2:** Association between dental insurance status with dental visiting behaviours and oral health status outcomes. Unadjusted proportion, adjusted proportions, and associated marginal effects are reported with 95% confidence intervals

	Dental visiting behaviours				Oral health status outcomes			
	Visiting the dentist in past 12 months ^a^		Visiting a dentist only for emergencies ^a^		Very good or excellent SROH ^a^		Tooth loss due to decay or gum disease ^b^	
	n = 42,553 ^a^		n = 42,553 ^a^		n = 42,553 ^a^		*n* = 29,426 ^b^	
Insurance Status	Unadjusted	Adjusted ^c^	Unadjusted	Adjusted ^c^	Unadjusted	Adjusted ^c^	Unadjusted	Adjusted ^d^
Yes	82.0 (81.1, 82.9)	79.4 (78.5, 80.4)	10.3 (9.6, 10.9)	13.2 (12.4, 14.0)	60.5 (59.4, 61.7)	57.9 (56.8, 59.0)	4.3 (3.7, 4.8)	5.7 (5.0, 6.4)
No	50.0 (48.4, 51.6)	56.6 (55.0, 58.2)	38.3 (36.7, 39.9	32.3 (30.8, 33.8)	44.1 (42.5, 45.7)	48.3 (46.7, 49.9)	8.0 (6.9, 9.0)	7.2 (6.2, 8.1)
Marginal Effects	32.0 (30.1, 33.8)	22.8 (20.9, 24.7)	−28.0 (−29.8, −26.3)	−19.1 (−20.9, −17.4)	16.4 (14.4, 18.3)	9.6 (7.6, 11.5)	−3.7 (−4.9, − 2.5)	−1.5 (− 2.7, −0.3)

^a^ Represents 11,682,112 Ontarians

^b^ Restricted to Ontarians who visited dentist in past 12 months, and have at least one of their own teeth at the time of survey completion

^c^ Adjusted for age, sex, dental insurance, income quintile, household education, self-perceived general health, geographic peer group, and having at least one of your own teeth

^d^ Adjusted for age, sex, dental insurance, income quintile, household education, self-perceived general health and geographic peer group

### Objective 2: the impact of insurance across income, education and age subgroups

Table 3 shows the adjusted ME of dental insurance on dental visiting behaviour estimated across income, education and age group strata. Across all covariate strata a greater proportion of individuals with insurance visited a dentist in the past 12 months compared to those without insurance. Dental insurance has variable impact on visiting a dentist by income quintile, such that we observe a larger ME for individuals in the lowest income quintile compared to the highest (ME lowest 27.2, 95%CI: 25.0–29.3; ME highest 17.9, 95%CI: 15.9–19.8). Across all strata individuals with insurance were consistently less likely to report visiting a dentist only for emergencies. Once again, there was a gradient in ME across income quintiles, with larger MEs in the lowest income quintile (− 27.2, 95%CI: − 29.5 to − 24.8) than the highest income quintile (− 11.5, 95%CI: − 13.2 to − 9.9). There was little variation in the ME of insurance across different levels of household education or age groups. The notable exception is that visiting a dentist among 12–17-year-olds has the smallest ME compared to other age groups (ME for 12–17-year-olds: 15.4, 95%CI: 13.3–17.5).

**Table 3:** Marginal effects of dental insurance on dental visiting behaviours. Analysis is shown for the study population and for income, education and age group strata. For each outcome, overall proportion, proportion stratified by dental insurance status, and marginal effect of having dental insurance are shown with 95% confidence intervals. Analysis is modelled in logistic regression and adjusted for age, sex, dental insurance, income quintile, household education, self-perceived general health, geographic peer group, and having at least one of your own teeth

	Visiting a dentist in past 12 months ^a^				Visiting a dentist only for emergencies ^a^			
	Overall Proportion	No dental insurance	Yes dental insurance	Marginal effect	Overall Proportion	No dental insurance	Yes dental insurance	Marginal effect
All	71.2 (70.4, 71.9)	56.6 (55.0, 58.2)	79.4 (78.5, 80.4)	22.8 (20.9, 24.7)	20.7 (20.0, 21.4)	32.3 (30.8, 33.8)	13.2 (12.4, 14.0)	−19.1 (− 20.9, − 17.4)
Income quintile								
Lowest	61.1 (59.0, 63.3)	43.1 (40.5, 45.7)	70.2 (68.0, 72.5)	27.2 (25.0, 29.3)	31.2 (29.2, 33.2)	49.1 (46.2, 51.9)	21.9 (19.9, 24.0)	−27.2 (−29.5, −24.8)
Lower middle	67.2 (65.3, 69.1)	50.3 (47.7, 52.9)	75.9 (73.9, 77.9)	25.6 (23.5, 27.7)	24.4 (22.7, 26.0)	39.7 (37.1, 42.3)	16.1 (14.6, 17.7)	−23.5 (−25.7, −21.4)
Middle	72.3 (70.7, 74.0)	56.9 (54.3, 59.5)	80.4 (78.9, 81.9)	23.5 (21.4, 25.6)	18.1 (16.5, 19.6)	30.2 (27.5, 32.9)	11.3 (10.0, 12.5)	−18.9 (−21.0, −16.9)
Upper middle	76.2 (74.6, 77.8)	62.2 (59.6, 64.9)	83.7 (82.3, 85.0)	21.5 (19.4, 23.5)	15.0 (13.7, 16.3)	25.3 (22.9, 27.7)	9.0 (8.0, 10.0)	−16.3 (−18.2, − 14.3)
Highest	81.6 (80.1, 83.1)	70.1 (67.6, 72.7)	88.0 (86.8, 89.1)	17.9 (15.9, 19.8)	10.1 (8.9, 11.3)	17.2 (15.1, 19.4)	5.7 (4.9, 6.5)	−11.5 (−13.2, −9.9)
Household Education								
< Than secondary	63.0 (60.1, 65.8)	46.0 (42.3, 49.7)	71.9 (69.1, 74.8)	25.9 (23.7, 28.2)	26.6 (23.7, 29.5)	41.2 (36.8, 45.7)	17.9 (15.4, 20.5)	−23.3 (−26.0, −20.6)
Secondary graduate	67.3 (65.4, 69.2)	51.3 (48.5, 54.1)	76.0 (74.2, 77.9)	24.7 (22.5, 26.9)	24.5 (22.8, 26.2)	38.3 (35.4, 41.1)	16.2 (14.6, 17.7)	−22.1 (−24.4, −19.9)
Some post secondary	69.4 (65.0, 73.8)	54.0 (48.1, 59.9)	77.9 (74.0, 81.9)	23.9 (21.2, 26.6)	24.0 (19.5, 28.4)	37.5 (31.0, 44.0)	15.7 (12.0, 19.4)	−21.8 (− 25.1, −18.4)
Post secondary graduate	72.7 (71.8, 73.6)	58.3 (56.6, 60.0)	80.8 (79.8, 81.8)	22.5 (20.6, 24.4)	19.1 (18.3, 19.9)	30.4 (28.8, 31.9)	11.9 (11.0, 12.7)	−18.5 (− 20.2, − 16.8)
Age								
12–17	83.4 (81.4, 85.4)	74.0 (70.8, 77.3)	89.5 (87.9, 91.0)	15.4 (13.3, 17.5)	11.9 (9.9, 13.9)	18.8 (15.4, 22.1)	6.8 (5.4, 8.1)	−12.0 (− 14.2, −9.8)
18–34	64.7 (63.2, 66.3)	48.2 (45.7, 50.6)	73.6 (71.9, 75.2)	25.4 (23.2, 27.5)	22.7 (21.3, 24.2)	35.3 (32.9, 37.7)	14.8 (13.4, 16.1)	−20.5 (− 22.5, − 18.5)
35–49	68.7 (66.9, 70.4)	53.2 (50.5, 55.9)	77.2 (75.6, 78.9)	24.0 (21.8, 26.2)	23.3 (21.5, 25.0)	36.0 (33.1, 39.0)	15.2 (13.7, 16.8)	−20.8 (− 23.0, − 18.6)
50–64	73.1 (71.4, 74.7)	59.1 (56.6, 61.6)	81.1 (79.6, 82.7)	22.0 (20.1, 24.0)	20.6 (19.3, 22.0)	32.2 (29.9, 34.5)	13.1 (11.9, 14.3)	−19.1 (− 21.0, − 17.1)
65+	76.0 (74.7, 77.2)	63.1 (61.2, 65.1)	83.6 (82.2, 84.9)	20.4 (18.8, 22.1)	18.9 (17.7, 20.0)	29.6 (27.7, 31.4)	11.8 (10.6, 12.9)	−17.8 (− 19.4, − 16.2)

a – 42,553 sample representing 11,682,112 Ontarians

Table 4 shows the adjusted ME of dental insurance on oral health outcomes across household income quintile, household education, and age group strata. While having insurance is associated with an increase in the proportion of people who report very good or excellent SROH, there was no substantial variation across subgroups. There was also a flatter gradient in the ME of insurance on the proportion of participants who reported tooth extraction due to decay or gum disease across income quintiles (ME highest quintile: -0.9, 95%CI: − 1.6 to − 0.2; ME lowest quintile: -2.5, 95%CI: − 4.4 to − 0.6).

**Table 4:** Marginal effects of dental insurance on oral health status outcomes. Analysis is shown for the study population and for income, education and age group strata. For each outcome, overall proportion, proportion stratified by dental insurance status, and marginal effect of having dental insurance are shown with 95% confidence intervals. Analysis is modelled in logistic regression and adjusted as described below

	Very good or excellent self-reported oral health ^a^				Tooth loss due to decay or gum disease ^b^			
	Overall Proportion	No dental insurance	Yes dental insurance	Marginal effect	Overall Proportion	No dental insurance	Yes dental insurance	Marginal effect
All	54.6 (53.8, 55.5)	48.3 (46.7, 49.9)	57.9 (56.8, 59.0)	9.6 (7.6, 11.5)	6.3 (5.8, 6.9)	7.2 (6.2, 8.1)	5.7 (5.0, 6.4)	−1.5 (−2.7, −0.3)
Income quintile								
Lowest	47.4 (45.2, 49.7)	40.9 (38.5, 43.3)	50.7 (48.3, 53.1)	9.8 (7.8, 11.8)	10.3 (8.4, 12.1)	11.8 (9.6, 14.1)	9.3 (7.3, 11.3)	−2.5 (−4.4, −0.6)
Lower middle	49.9 (47.8, 52.0)	43.3 (41.0, 45.6)	53.2 (50.9, 55.5)	9.9 (7.9, 11.9)	8.1 (6.5, 9.6)	9.3 (7.3, 11.3)	7.3 (5.8, 8.8)	−2.0 (−3.6, −0.4)
Middle	54.4 (52.5, 56.3)	47.9 (45.5, 50.3)	57.7 (55.8, 59.7)	9.8 (7.8, 11.8)	4.8 (3.8, 5.9)	5.6 (4.3, 7.0)	4.3 (3.4, 5.3)	−1.3 (−2.3, −0.3)
Upper middle	58.5 (56.6, 60.3)	52.1 (49.6, 54.6)	61.8 (59.9, 63.6)	9.7 (7.7, 11.7)	4 (3.1, 4.8)	4.6 (3.4, 5.8)	3.6 (2.8, 4.3)	−1.1 (−2.0, −0.2)
Highest	63.1 (61.2, 65.0)	57.0 (54.4, 59.5)	66.3 (64.4, 68.2)	9.3 (7.4, 11.3)	3.2 (2.5, 3.9)	3.7 (2.8, 4.7)	2.9 (2.2, 3.5)	−0.9 (−1.6, − 0.2)
Household Education								
< Than secondary	46.2 (43.1, 49.3)	39.7 (36.4, 43.0)	49.4 (46.1, 52.6)	9.6 (7.7, 11.6)	8.8 (6.7, 11.0)	9.8 (7.4, 12.3)	7.8 (5.8, 9.9)	−2.1 (−3.8, −0.5)
Secondary graduate	50.9 (48.6, 53.3)	44.4 (41.7, 47.2)	54.2 (51.7, 56.6)	9.7 (7.7, 11.7)	7.8 (6.2, 9.4)	8.8 (6.8, 10.8)	7 (5.5, 8.4)	−1.9 (−3.5, −0.4)
Some post secondary	47.8 (42.6, 53.1)	41.4 (36.0, 46.8)	51.1 (45.7, 56.4)	9.7 (7.7, 11.7)	8.8 (5.0, 12.6)	9.9 (5.6, 14.3)	7.9 (4.5, 11.3)	−2.1 (−4.0, −0.2)
Post secondary graduate	56.1 (55.1, 57.1)	49.8 (48.1, 51.4)	59.4 (58.2, 60.6)	9.7 (7.7, 11.6)	5.5 (4.9, 6.1)	6.3 (5.4, 7.2)	4.9 (4.2, 5.7)	−1.4 (−2.5, −0.3)
Age								
12–17	56.1 (53.6, 58.7)	49.8 (46.8, 52.9)	59.4 (56.8, 62.0)	9.5 (7.6, 11.5)	1.0 (0.4, 1.6)	1.1 (0.4, 1.8)	0.8 (0.3, 1.4)	−0.3 (−0.5, 0)
18–34	55.8 (54.1, 57.5)	49.5 (47.3, 51.7)	59.1 (57.3, 60.9)	9.6 (7.6, 11.5)	4.0 (3.1, 5.0)	4.6 (3.4, 5.9)	3.6 (2.7, 4.5)	−1.1 (−1.9, −0.2)
35–49	54.8 (52.8, 56.8)	48.5 (46.0, 50.9)	58.0 (56.0, 60.1)	9.6 (7.6, 11.6)	6.4 (5.0, 7.9)	7.4 (5.6, 9.1)	5.7 (4.3, 7.1)	−1.6 (−2.9, −0.4)
50–64	51.2 (49.3, 53.0)	44.7 (42.4, 47.0)	54.4 (52.4, 56.4)	9.6 (7.7, 11.6)	9.1 (7.7, 10.4)	10.3 (8.4, 12.2)	8.1 (6.8, 9.5)	−2.2 (−4.0, −0.5)
65+	56.8 (55.1, 58.5)	50.5 (48.5, 52.5)	60.0 (58.1, 62.0)	9.5 (7.6, 11.5)	7.8 (6.6, 8.9)	8.9 (7.4, 10.3)	6.9 (5.7, 8.2)	−1.9 (−3.4, −0.5)

a – 42,553 sample representing 11,682,112 Ontarians. Analyses adjusted for age, sex, dental insurance, income quintile, household education, self-perceived general health, geographic peer group, and having at least one of your own teeth

b – 29,426 to 29,472 sample representing only Ontarians who visited dentist in past 12 months, and have at least one of their own teeth at the time of survey completion. Analysis is adjusted for age, sex, dental insurance, income quintile, household education, self-perceived general health and geographic peer group

## Discussion

Our study revealed that individuals with dental insurance had more favourable oral health-related outcomes overall and across all income, education and age groups, after adjusting for covariates. Larger ME for dental insurance were observed for visiting the dentist for lower income compared to higher income quintiles, showing that the most financially disadvantaged groups would likely benefit the most if universal dental coverage were implemented. This is an example of proportionate universalism, which is generally considered very positive, given that everyone receives the intervention, but benefits accrue in the least well off [33].

Our study adds to the large body of literature supporting the role of insurance in accessing dental care [1, 19, 22, 34]. For example, in the United States, Choi compared dental visiting behaviour among low-income parents in States with and without Medicaid coverage, using a similar cross-sectional survey design as our study. Their results revealed that expanded Medicaid dental services increased the probability of a dental visit in the past 12 months by 16.4 to 22.0% amongst parents who have an income under $10,000 [21]. Our findings suggest that being publicly or privately insured increases the proportion visiting the dentist by 25.0 to 29.3% amongst those in the lowest income quintile. Work conducted by Baiker and colleagues in Oregon, United States further corroborates our findings. Based on an experimental design, these authors demonstrated that randomly allocating Medicaid coverage for emergency dental services to individuals increases the use of these services and reduces unmet dental care needs [35]. Conversely, another study from Oregon shows that eliminating Medicaid dental benefits in a proportion of enrollees results in more unmet needs and lower probability of annual dental check-ups amongst those who lost their benefits [36].

The World Health Organization advocates for universal dental coverage, with special attention to ensure that the most vulnerable population groups are able to access the care they need [17]. Our findings suggest that implementing a universal dental insurance program in Ontario could improve access to dental care and oral health-related outcomes. Improving access through universal dental coverage could reduce the use of hospital emergency departments and physician offices for non-traumatic dental problems (i.e. toothache), issues that have received significant policy attention in Canada [37–46]. Additionally, depending on the extent of the insured service package and individual needs, insurance may be able to alleviate financial burdens among the working poor and middle income groups as well [13, 47, 48].

The impetus for creating a universal dental care policy is developing in Canada. There are numerous routes that can be taken to achieve this, and the suggestions provided here are not exhaustive (see [23, 24] for more information). Dental care coverage could be implemented through a single-payer system similar to Canada’s broader health care system. Alternatively, compulsory insurance policies, as found in countries such as Switzerland and the Netherlands, could be implemented to ensure that all citizens are covered by some form of public or private insurance. In this type of model, residents are automatically enrolled in a government plan unless they choose an alternative government-approved private plan. Finally, governments may opt to expand the eligibility criteria for existing publicly funded dental programs to ensure that the most financially vulnerable groups have access to dental insurance coverage. For now, increasing support for this group may have the biggest impact.

Our results should be considered in the context of their limitations. We did not have detailed information about insurance coverage, so we are not able to determine to what extent insurance comprehensiveness and quality affects our results. Self-reported survey data is subject to various biases. Individuals may answer questions incorrectly due to recall error or give socially acceptable answers, or they may choose to skip a question they feel uncomfortable answering. In terms of sampling, the CCHS limits its eligibility criteria to those who have a telephone [28]. Statistics Canada accounts for missing income data using a complex methodology of near neighbor imputation [30], and we accounted for remaining missing data using multiple imputations. Using these methods, bias from missing data would arguably be reduced.

The large sample size in our study allows us to make valuable population-level estimates in Ontario, Canada’s most populated province. Our findings are most applicable in an Ontario context. Canadian provinces have similar financing schemes and dental care systems, and may possibly show similar results to this study, where comparable analyses are conducted. We would expect insurance to have a strong impact on dental care across all Canadian jurisdictions.

## Conclusion

Our analyses contribute to the policy debate regarding universal dental coverage. We demonstrate that even after adjustment for sociodemographic characteristics, those who have insurance report visiting a dentist and excellent or very good oral health more than those who do not. In fact, the positive impacts of insurance are present across all groups. Insurance coverage for all Canadians will likely demonstrate the benefits of proportionate universalism, improving equity in access to dental care and oral health-related outcomes across the country.

## Supplementary information

**Additional file 1.** Unadjusted proportion of individuals in each sociodemographic group with each outcome of interest, including dental visiting behaviours and oral health status outcomes. Proportions with 95% confidence intervals are reported.

**Additional file 2.** Marginal effects (95% confidence intervals) for insurance, income quintile, educations and age group in adjusted logistic regression models with each of the outcomes of interest. Values represent a percentage point change from the baseline reference group.

## Data Availability

The Canadian Community Health Surveys are made available to students and staff affiliated with the University of Toronto through the Computing by the Humanities and Social Sciences (CHASS) data centre. Requests and further information on accessing the dataset can be obtained here: https://mdl.library.utoronto.ca/research/help

## Abbreviations

CCHS: Canadian Community Health Survey
CI: Confidence interval
MAR: Missing at random
ME: Marginal effects
MICE: Multiple imputation with chained equations
OECD: Organization for Economic Cooperation and Development
SROH: Self-reported oral health
