# Correction to: Providing dental insurance can positively impact oral health outcomes in Ontario

**DOI:** 10.1186/s12913-021-06237-2

**Published:** 2021-03-12

**Authors:** Nevena Zivkovic, Musfer Aldossri, Noha Gomaa, Julie W. Farmer, Sonica Singhal, Carlos Quiñonez, Vahid Ravaghi

**Affiliations:** 1 Dental Public Health, Faculty of Dentistry, University of Toronto, Toronto, Canada; 2 School of Dentistry, University of Birmingham, Birmingham, England

**Correction to: BMC Health Serv Res 20, 124 (2020)**

**https://doi.org/10.1186/s12913-020-4967-3**

Following publication of the original article [1], the authors would like to add some information in the Competing interests section.

The updated content in the Competing interests is shown below:

Carlos Quiñonez receives consulting income for dental care related issues from Green Shield Canada. All other authors declare no competing interests.

The original article has been corrected.
