# Supplementary material for: Providing dental insurance can positively impact oral health outcomes in Ontario
Source: BMC Health Serv Res. 2020 Feb 17;20:124. doi: 10.1186/s12913-020-4967-3 (PMC7027064; doi:10.1186/s12913-020-4967-3)
Supplement: Supplementary file 1 — Additional file 1. Unadjusted proportion of individuals in each sociodemographic group with each outcome of interest, including dental visiting behaviours and oral health status outcomes. Proportions with 95% confidence intervals are reported. [file 12913_2020_4967_MOESM1_ESM.docx]

**Additional File 1**: Unadjusted proportion of individuals in each sociodemographic group with each outcome of interest, including dental visiting behaviours and oral health status outcomes.

|  | **Dental visiting behaviours** | | **Oral health status outcomes** | |
| --- | --- | --- | --- | --- |
|  | **Visiting a dentist in past 12 months ^a^** | **Visits a dentist only for emergencies ^a^** | **Very good or excellent SROH ^a^** | **Tooth loss due to decay or gum disease ^b^** |
| **Insurance** |  |  |  |  |
| No | 50.0 (48.4-51.6) | 38.3 (36.7-39.9) | 44.1 (42.5-45.7) | 8.0 (6.9-9.0) |
| Yes | 82.0 (81.1-82.9) | 10.3 (9.6-10.9) | 60.5 (59.4-61.7) | 4.3 (3.7-4.8) |
| **Income quintile** |  |  |  |  |
| Lowest | 52.1 (49.7-54.4) | 38.8 (36.5-41.1) | 41.4 (39.1-43.7) | 10 (8.3-11.7) |
| Lower middle | 63.8 (61.7-65.9) | 26.2 (24.4-28.1) | 47.6 (45.5-49.8) | 7.9 (6.3-9.4) |
| Middle | 73.4 (71.7-75.2) | 16 (14.5-17.4) | 55.6 (53.5-57.6) | 4.3 (3.4-5.2) |
| Upper middle | 80.4 (78.9-81.8) | 10.9 (9.8-12.1) | 62.1 (60.2-64.0) | 3.3 (2.6-4.0) |
| Highest | 86.5 (85.3-87.7) | 6.2 (5.4-7.0) | 68.3 (66.5-70.1) | 2.7 (2.1-3.2) |
| **Education** |  |  |  |  |
| <Secondary | 42 (38.8-45.2) | 49.0 (45.5-52.5) | 32.7 (29.9-35.5) | 14.8 (11.6-18.0) |
| Secondary graduate | 60.2 (57.8-62.7) | 30.8 (28.4-33.2) | 45.6 (43.1-48.1) | 8.5 (6.7-10.3) |
| Some post secondary | 63.1 (57.5-68.6) | 27.6 (22.5-32.7) | 43.6 (38.1-49.1) | 7.8 (4.2-11.4) |
| Post secondary graduate | 75.5 (74.5-76.4) | 15.3 (14.5-16.1) | 58.6 (57.6-59.7) | 4.2 (3.8-4.7) |
| **Age group** |  |  |  |  |
| 12-17 | 86.9 (85.1-88.8) | 8.1 (6.4-9.9) | 59.7 (57.0-62.4) | 0.8 (0.3-1.3) |
| 18-34 | 66.7 (64.9-68.5) | 19.5 (18.1-21) | 58.6 (56.8-60.4) | 3.2 (2.4-4.0) |
| 35-49 | 74.0 (72.0-76.0) | 17.5 (15.8-19.2) | 57.7 (55.6-59.9) | 4.6 (3.5-5.6) |
| 50-64 | 75.2 (73.5-77.0) | 17.7 (16.3-19.1) | 51.2 (49.2-53.2) | 7.4 (6.3-8.4) |
| 65+ | 61.2 (59.6-62.7) | 30.5 (28.9-32.0) | 48.9 (47.3-50.5) | 8.4 (7.2-9.5) |

a – 42,553 sample representing 11,682,112 Ontarians

b – 29,426 to 29,472 sample representing only Ontarians who visited dentist in past 12 months, and have at least one of their own teeth at the time of survey completion
