# Supplementary material for: Providing dental insurance can positively impact oral health outcomes in Ontario
Source: BMC Health Serv Res. 2020 Feb 17;20:124. doi: 10.1186/s12913-020-4967-3 (PMC7027064; doi:10.1186/s12913-020-4967-3)
Supplement: Supplementary file 2 — Additional file 2. Marginal effects (95% confidence intervals) for insurance, income quintile, educations and age group in adjusted logistic regression models with each of the outcomes of interest. Values represent a percentage point change from the baseline reference group. [file 12913_2020_4967_MOESM2_ESM.docx]

**Additional File 2.** Marginal effects (95% confidence intervals) for insurance, income quintile, educations and age group in adjusted logistic regression models with each of the outcomes of interest. Values represent a percentage point change from the baseline reference group.

|  | **Dental visiting behaviours** | | **Oral health status outcomes** | |
| --- | --- | --- | --- | --- |
|  | **Visiting a dentist in past 12 months ^a^** | **Visiting a dentist only for emergencies ^a^** | **Very good or excellent SROH ^a^** | **Tooth removed due to decay or gum disease ^b^** |
| **Insurance** |  |  |  |  |
| No | Reference | Reference | Reference | Reference |
| Yes | 22.8 (20.9, 24.7) | -19.1 (-20.9, -17.4) | 9.6 (7.6, 11.5) | -1.5 (-2.7, -0.3) |
| **Income quintile** |  |  |  |  |
| Lowest | Reference | Reference | Reference | Reference |
| Lower middle | 6.1 (3.3, 8.9) | -6.9 (-9.4, -4.3) | 2.4 (-0.6, 5.4) | -2.2 (-4.6, 0.3) |
| Middle | 11.2 (8.5, 14.0) | -13.2 (-15.7, -10.6) | 7.0 (4.0, 10.0) | -5.4 (-7.6, -3.2) |
| Upper middle | 15.1 (12.3, 17.8) | -16.3 (-18.7, -13.8) | 11.0 (8.1, 14.0) | -6.3 (-8.4, -4.2) |
| Highest | 20.5 (17.7, 23.3) | -21.1 (-23.6, -18.7) | 15.7 (12.6, 18.8) | -7.1 (-9.1, -5.0) |
| **Education** |  |  |  |  |
| <Secondary | Reference | Reference | Reference | Reference |
| Secondary graduate | 4.4 (1.1, 7.7) | -2.1 (-5.4, 1.2) | 4.7 (1.1, 8.4) | -1.2 (-3.8, 1.5) |
| Some post secondary | 6.5 (1.2, 11.7) | -2.6 (-8.0, 2.7) | 1.7 (-4.3, 7.6) | -0.2 (-4.6, 4.2) |
| Post secondary graduate | 9.7 (6.7, 12.8) | -7.5 (-10.5, -4.4) | 10.0 (6.7, 13.2) | -3.4 (-5.7, -1.1) |
| **Age group** |  |  |  |  |
| 12-17 | Reference | Reference | Reference | Reference |
| 18-34 | -18.6 (-21.2, -16.1) | 10.8 (8.3, 13.3) | -0.3 (-3.4, 2.7) | 3.1 (1.9, 4.2) |
| 35-49 | -14.7 (-17.4, -12.0) | 11.3 (8.7, 14.0) | -1.3 (-4.5, 1.9) | 5.4 (3.9, 7.0) |
| 50-64 | -10.3 (-12.9, -7.7) | 8.7 (6.3, 11.2) | -5.0 (-8.2, -1.8) | 8.1 (6.6, 9.5) |
| 65+ | -7.4 (-9.9, -5.0) | 7.0 (4.6, 9.4) | 0.6 (-2.5, 3.8) | 6.8 (5.5, 8.1) |

a – 42,553 sample representing 11,682,112 Ontarians. Analyses adjusted for age, sex, dental insurance, income quintile, household education, self-perceived general health, geographic peer group, and having at least one of your own teeth.

b – 29,426 to 29,472 sample representing only Ontarians who visited dentist in past 12 months, and have at least one of their own teeth at the time of survey completion. Analysis is adjusted for age, sex, dental insurance, income quintile, household education, self-perceived general health and geographic peer group.
